# Sensitivity of Human Intrahepatic Cholangiocarcinoma Subtypes to Chemotherapeutics and Molecular Targeted Agents: A Study on Primary Cell Cultures

**DOI:** 10.1371/journal.pone.0142124

**Published:** 2015-11-16

**Authors:** Alice Fraveto, Vincenzo Cardinale, Maria Consiglia Bragazzi, Felice Giuliante, Agostino Maria De Rose, Gian Luca Grazi, Chiara Napoletano, Rossella Semeraro, Anna Maria Lustri, Daniele Costantini, Lorenzo Nevi, Sabina Di Matteo, Anastasia Renzi, Guido Carpino, Eugenio Gaudio, Domenico Alvaro

**Affiliations:** 1 Department of Medico-Surgical Sciences and Biotechnologies, Sapienza University of Rome, Rome, Italy; 2 Health Science, University of Rome “Foro Italico”, Rome, Italy; 3 SAIMLAL, Sapienza, University of Rome, Rome, Italy; 4 Surgery, Hepatobiliary Unit, Catholic University of the Sacred Heart School of Medicine, Rome, Italy; 5 Experimental Medicine, Sapienza University of Rome, Rome, Italy; 6 Hepato-Biliary Surgery, Regina Elena National Cancer Institute, Rome, Italy; Texas A&M Health Science Center, UNITED STATES

## Abstract

We investigated the sensitivity of intrahepatic cholangiocarcinoma (IHCCA) subtypes to chemotherapeutics and molecular targeted agents. Primary cultures of mucin- and mixed-IHCCA were prepared from surgical specimens (N. 18 IHCCA patients) and evaluated for cell proliferation (MTS assay) and apoptosis (Caspase 3) after incubation (72 hours) with increasing concentrations of different drugs. *In vivo*, subcutaneous human tumor xenografts were evaluated. Primary cultures of mucin- and mixed-IHCCA were characterized by a different pattern of expression of cancer stem cell markers, and by a different drug sensitivity. Gemcitabine and the Gemcitabine-Cisplatin combination were more active in inhibiting cell proliferation in mixed-IHCCA while Cisplatin or Abraxane were more effective against mucin-IHCCA, where Abraxane also enhances apoptosis. 5-Fluoracil showed a slight inhibitory effect on cell proliferation that was more significant in mixed- than mucin-IHCCA primary cultures and, induced apoptosis only in mucin-IHCCA. Among Hg inhibitors, LY2940680 and Vismodegib showed slight effects on proliferation of both IHCCA subtypes. The tyrosine kinase inhibitors, Imatinib Mesylate and Sorafenib showed significant inhibitory effects on proliferation of both mucin- and mixed-IHCCA. The MEK 1/2 inhibitor, Selumetinib, inhibited proliferation of only mucin-IHCCA while the aminopeptidase-N inhibitor, Bestatin was more active against mixed-IHCCA. The c-erbB2 blocking antibody was more active against mixed-IHCCA while, the Wnt inhibitor, LGK974, similarly inhibited proliferation of mucin- and mixed-IHCCA. Either mucin- or mixed-IHCCA showed high sensitivity to nanomolar concentrations of the dual PI3-kinase/mTOR inhibitor, NVP-BEZ235. *In vivo*, in subcutaneous xenografts, either NVP-BEZ235 or Abraxane, blocked tumor growth. In conclusion, mucin- and mixed-IHCCA are characterized by a different drug sensitivity. Cisplatin, Abraxane and the MEK 1/2 inhibitor, Selumetinib were more active against mucin-IHCCA while, Gemcitabine, Gemcitabine-Cisplatin combination, the c-erbB2 blocking antibody and bestatin worked better against mixed-IHCCA. Remarkably, we identified a dual PI3-kinase/mTOR inhibitor that both *in vitro* and *in vivo*, exerts dramatic antiproliferative effects against both mucin- and mixed-IHCCA.

## Introduction

Cholangiocarcinoma (CCA) arises from the neoplastic transformation of the epithelial cells lining the intrahepatic (IH) and extrahepatic (EH) bile ducts and/or the peribiliary glands [1]. CCA prognosis is very bad with an average survival of less than twelve months and this because surgical resection, the only effective treatment, is possible in less than half of the patients [1]. Unfortunately, diagnosis often occurs in an advanced stage where only palliative treatments are applicable. CCA is highly resistant to chemotherapeutics and the current standard of care, Gemcitabine/Cisplatin combination, ensures only few months improved survival [1]. Cancer stem cells (CSCs) are particularly prone to be involved in the carcinogenic process due to their particular biological features [2–4]. According to recent advances, CSCs confer resistance to chemotherapeutics other than being responsible for tumor recurrence. Therefore, CSCs have been the focus of extensive investigations as cell of origin of different cancers and as therapeutic targets [2–4]. We have recently demonstrated that human CCA is highly enriched in CSCs and this could be one of the reason for resistance to pharmacological treatments [5]. According to recent advances [1], CCA is classified as intrahepatic (IHCCA), perihilar and distal. The IHCCA is comprised of two different subtypes [5, 6] that are a mucin subtype constituted by pure mucin-secreting cells and displaying similarities with perihilar-CCA and, a mixed form comprising areas of hepatocytic differentiation and neoplastic ductular reaction. This distinction reflects the cells of origin since pathology and molecular analyses indicate that mucin-CCAs derive from the mucin-secreting epithelium lining large ducts or from PBGs [6]. By contrast, the mixed form derives from cuboidal non-mucin-secreting cells lining bile ductules or canals of Hering. Large morpho-pathological differences exist between mucin- and mixed-IHCCA, including the CSC profile [5]; nonetheless, no information exists on whether the two subtypes display a different sensitivity to antineoplastic treatments. The aim of our study was to evaluate the *in vitro* sensitivity of human CCA primary cell cultures, prepared from mucin- and mixed-CCA, to chemotherapeutics and molecular targeted agents.

## Materials and Methods

Genistein, Cyclopamine and 5-Fluorouracil (5-Fu) were purchased from Sigma-Aldrich Co (St Louis, MO, USA). Cisplatin, Gemcitabine, Vismodegib, LY2940680, Imatinib mesylate, Bestatin, NVP-BEZ235, AZD6244 (Selumetinib), MK2206 and LGK974 were purchased from Selleck Chemicals (Houston, TX, USA). Cetuximab was purchased from Merck Serono (Rome, Italy). The c-ErbB2 blocking antibody was obtained from Spring Bioscience Corporation (Pleasanton, CA, USA). Abraxane (Nab-Paclitaxel) was obtained from Abraxis BioScience (Los Angeles, CA, USA).

### Human CCA Specimens and Cell Cultures

The use of human materials has been approved by our local Institutional Review Board. Specimens of human IHCCA were obtained from patients submitted to surgical resection and specifically: 18 patients with IH-CCA presenting as a single mass lesion within the liver. Patient characteristics were detailed in Table 1.

**Table 1:** Patients characteristics.

	Mixed-IHCCA (N = 9)	Mucin-IHCCA (N = 9)
Age (yrs, mean ± SD)	70±8	68±10
Age (yrs, range)	59–83	50–81
Female/Male	5/4	5/4
Putative risk factors*		
Cirrhosis	4	3
HCV-Ab positive	3	3
Diabetes	2	3
Obesity	2	2
Gallstones	3	3
No risk factor	4	4

*according to ref. # 1.

Human IHCCA samples were classified as mixed- or mucin-IHCCA by morphologic criteria and Pas staining, according to Komuta M. et al [6]. Patients characteristics CCA samples were subjected to mechanical and enzymatic dissociation with type IV collagenase (100U/ml) (Sigma Aldrich, Milan, Italy) at 37°C for 12–14 hours. Cells were plated in hormonally supplemented medium consisting of DMEM with high glucose/DMEM:F12 mixture (1:1) (Gibco/BRL, Life Technologies, Italia srl., Milan, Italy) supplemented with 1.8 x 10^−4^ mol/L adenine, 5 μg/ml insulin, 5 μg/ml transferrin, 2 x 10^−9^ mol/L triiodothyronine, 1.7 x 10^−6^ mol/L hydrocortisone, 1.0 x 10^−6^ mol/L human epidermal growth factor, 5.5 x 10^−6^ mol/L epinephrine (Sigma-Aldrich, Milan, Italy), 10% fetal bovine serum (Gibco/BRL, Life Technologies, Milan, Italy), 100 U/ml of penicillin, and 100 μg/ml of streptomycin, at 37°C in a humidified atmosphere of 5% CO^2^ in air. Primary cell cultures were maintained at 37°C in a humidified atmosphere of 5% CO^2^ in air. The different drugs were tested after 20–30 passages.

### Characterization of Primary CCA Cell Cultures by Flow Cytometry (FC), Immunohistochemistry/Immunofluorescence (IHC/IF) and RT-PCR

Primary IHCCA cell cultures were investigated by FC, at passages 20–30, for the expression of CSC surface markers by using the following antibodies: PE-mouse anti-human CD13 (BD Pharmigen, Milan, Italy), CD90-FITC human, CD133-APC human, CD45-PE human, EpCAM-FITC human (Miltenyi Biotec, Milan, Italy), anti-LGR5 mouse mAb PE coniugate (Origene, Unimed Scientifica, Rome, Italy). The fluorescence threshold between negative and positive cells was set on the basis of the reactivity of appropriate non-specific fluorochrome-conjugated isotypical controls. At, least 5 X 10^5^ cells were analyzed using a FACS Diva software (BD).

For IHC/IF, semi-confluent cultures were generated on four-chamber slides (NUNC multiwell plates, Unimed Scientific, Italy). The medium was removed, and cells were fixed in 10% buffered formalin for 10 min at room temperature. Cells were rinsed twice with PBS buffer for 2 min, blocked and incubated 1 hour with the following antibodies: Vimentin mouse monoclonal IgG1 (sc-32322, Santa Cruz Biotechnology), α-SMA mouse monoclonal IgG1(M0851, Dako), E-Cadherin mouse monoclonal IgG1 (sc-21791, Santa Cruz Biotechnology), CD326/EpCAM mouse monoclonal IgG1 (sc-59782, Santa Cruz Biotechnology), CD133/PROM1 mouse monoclonal IgG1 (TA309943, Origene), LGR5 rabbit polyclonal IgG (TA301323,Origene), cytokeratin-19 (CK-19) sc-6278 CK-19 mouse monoclonal IgG2a (Santa Cruz Biotechnology), Interleukin 6 (IL6) mouse monoclonal IgG2a (ab9324, Abcam) at room temperature. After rinsing twice with PBS for 2 min, cells were incubated for 40 min at room temperature with secondary biotinylated antibody (Vector laboratories,DBA Italy) rinsed twice with PBS and then incubated with Vectastain ABC reagent (Vector laboratories, DBA Italy) for 20 min. Diaminobenzidine (DAB substrate kit, Vector laboratories, DBA Italy) was used as substrate, and sections were counterstained with hematoxylin. Slides were examined by DM 2000 Light and/or Fluorescence Microscopy (Leica Microsystems, Italy) equipped with a DFC450 C Videocam (Leica Microsystems,Italy). For RT-PCR, cell cultures were extracted for total RNA by using the TRI REAGENT^TM^ (Sigma-Aldrich, St Louis, MO, USA) and 1-bromo-3-chloropropane in substitution of chloroform. The isolated RNA was dissolved in 55 μl of RNase-free water. RNA quality and quantity was controlled by the Experion Automated Electrophoresis System equipped with the RNA StSens Analysis Chip (Bio-Rad Laboratories, Hercules, CA, USA). The reverse transcription primed by the random hexamer (Invitrogen s.r.l., S. Giuliano Milanese, Italy) was conducted in a 20 μL volume with an amount of 2.5 μg of total RNA and the M-MLV reverse transcriptase (Invitrogen s.r.l.) according the manufacturer's directions. Gene expression was determined by Real-Time PCR with a MX3000P instrument (Agilent, La J olla, CA, USA) using the averaged cycle threshold (Ct) automatically computed by the built-in software from three replicas of each sample. PCR amplifications were conducted into a volume of 25 μl, with 1.0 μl of cDNA template, 12.5 μl of 2x SYBR Green Brilliant QPCR Master Mix (Stratagene), 3 pmoles each of upstream and downstream primer for the gene analyzed, and 0.3 μl of diluted reference dye (ROX at a final concentration 30 nM). All real-time PCR amplifications were conducted with the cycling program: 10 min at 95°C followed by 40 cycles (30 sec at 95°C, 30 sec at 58°C, 30 sec at 72°C). The fluorescence detection was performed during the extension step of each cycle. The following genes of interest (GOI) were measured: Vimentin, CD13, CD90, CD133, EpCAM, and LGR5. All expression levels were normalized to the expression of GAPDH housekeeping gene. Table 2 shows the details of primers used in the study.

**Table 2:** Sequences of primer pairs (sense and antisense, respectively) used for amplifying the genes of interest (GOI) and the internal reference gene (GAPDH) used for their nor Primer designed by the PROBEFINDER software (https://www.roche-applied-science.com/sis/rtpcr/upl/index.jsp).

Gene Messenger	Primer Pair (5’->3’)	Length (nt)	Amplicon (bp)	Origin
GAPDH NM_002046	AGCCACATCGCTCAGACAC GCCCAATACGACCAAATCC	19 19	66	1
CD13 NM_001150	CAGTGACACGACGATTCTCC CCTGTTTCCTCGTTGTCCTT	20 20	76	1
CD44 NM_000610	TGCCGCTTTGCAGGTGTAT GGCCTCCGTCCGAGAGA	19 17	65	2
CD90 NM_006288	AGGACGAGGGCACCTACAC GCCCTCACACTTGACCAGTT	19 20	107	1
CD133 NM_002354	CCTGGGGCTGCTGTTTATTA ATCACCAACAGGGAGATTGC	20 20	161	1
EpCam NM_002354	ATAACCTGCTCTGAGCGAGTG TGAAGTGCAGTCCGCAAACT	21 20	104	3
VIMENTIN NM_003380	CTGCCAACCGGAACAATGA GTACTCAGTGGACTCCTGCTTT	19 22	56	3
LGR5 NM_003667	CTTCCAACCTCAGCGTCTTC TTTCCCGCAAGACGTAACTC	20 20	118	3

### *In Vitro* Sensitivity to Chemotherapeutics and Molecular Targeted Agents

Sensitivity to chemotherapeutics and molecular targeted agents was tested by evaluating cell proliferation or apoptosis in primary cell cultures exposed to increasing concentrations of different drugs. Drugs were prepared as a stock solution in DMSO and then diluted (> 1: 10,000) in the culture medium at the desired final concentration; the same amount of DMSO was added in controls. Proliferation was evaluated by MTS assay (CellTiter 96 Aqueous One Solution, PROMEGA, Milan, Italy). A total of 5x10^3^ cells were seeded into 96-well plates in 100 μL of culture medium. After 24 hours the medium was replaced with fresh culture medium containing increasing concentrations of the tested drug and then, after 72 hours, the MTS assay was performed. Results were expressed as % changes with respect to controls considered equal to 100. Apoptosis was evaluated by Caspase-3 Kit (SIGMA ALDRICH, Milan, Italy) by following instructions of the vendor. A total of 5x10^5^ cells were plated into flasks in 20 mL of culture medium. After 24 hours the medium was replaced with fresh culture medium containing a determined concentration of the different drugs; we tested the concentration that determined a significant inhibition of cell proliferation at the MTS assay. Apoptosis was detected after 72 hours and expressed as ratio between casapse-3 activity measured in drug-treated and control cells.

### *In Vivo* Sensitivity of Human Subcutaneous Xenografts to NVP-BEZ-235 and Abraxane

Male NOD/SCID mice, 4–6 weeks old, purchased from Charles River (Italy) were maintained under standard conditions and cared according to our institutional guidelines for animal care. As previously described [5], CD13+ and CD133+ spheroids were prepared from human mucin- or mixed-IHCCA primary cultures, suspended in culture medium/Matrigel mixture (1:1 volume) and injected (approximately 10,000 cells) subcutaneously into mid-abdominal areas. We used CD13+ and CD133+ spheroids since in the previous study [5], these CSC subpopulations showed the highest tumorigenic potential in terms of xenograft generation. Tumor xenograft formation was followed by macroscopic inspection. After fifteen days, when the tumor volume was about 500 mm^3^, mice were treated by gavage with NVP-BEZ235 (50 mg/Kg in PBS, three times a week) and Abraxane (10mg/Kg in PBS, twice a week) for two weeks. Control mice received PBS only. The health of all mice was monitored daily throughout the study. Main criteria used to assess mice health were the evaluation of body weight and consumption of food and water, other than the essentials for assessing mouse health as described by Burkholder et al. [7] Animal welfare was carefully ensured constantly by experienced operators every day. Every steps to avoid suffering were realized. Mice were then killed by cervical dislocation. The xenografts were removed after the death of the animal for histology.

### Ethics Statements

The research protocol was reviewed and approved by the *Ethics Committee of Hospital Policlinico Umberto I of Rome/Sapienza University of Rome* (full name of the board/committee; Prot. May 2014), and was conducted according to the principles expressed in the Declaration of Helsinki. Subjects have been properly instructed and have indicated that they consent to participate by signing the appropriate informed consent paperwork.

The experiment on animals was carried out in strict accordance with the recommendations in the Guide for the Care and Use of Laboratory Animals of the European Commission. The protocol was reviewed and approved by the *Ethics Committee of Hospital Policlinico Umberto I of Rome/Sapienza University of Rome* (full name of the board/committee; Prot. May 2014). Animal welfare was carefully ensured constantly by experienced operators every day. Mice were then killed by cervical dislocation. All efforts were made to minimize suffering of the animals along all the duration of their life and during the sacrifice.

The processing was compliant with Good Manufacturing Practice.

### Statistical Analysis

Data are presented as arithmetic mean ± S.D. Statistical analysis was conducted using the paired or unpaired Student’s *t*-test as appropriate or the analysis of the variance (ANOVA) when multiple comparisons were performed. A p value < 0.05 was considered statistically significant. The half maximal inhibitory concentration (IC_50_) values were calculated by nonlinear regression analysis.

## Results

### Characterization of Primary Cultures of Human CCA

**Fig 1:**
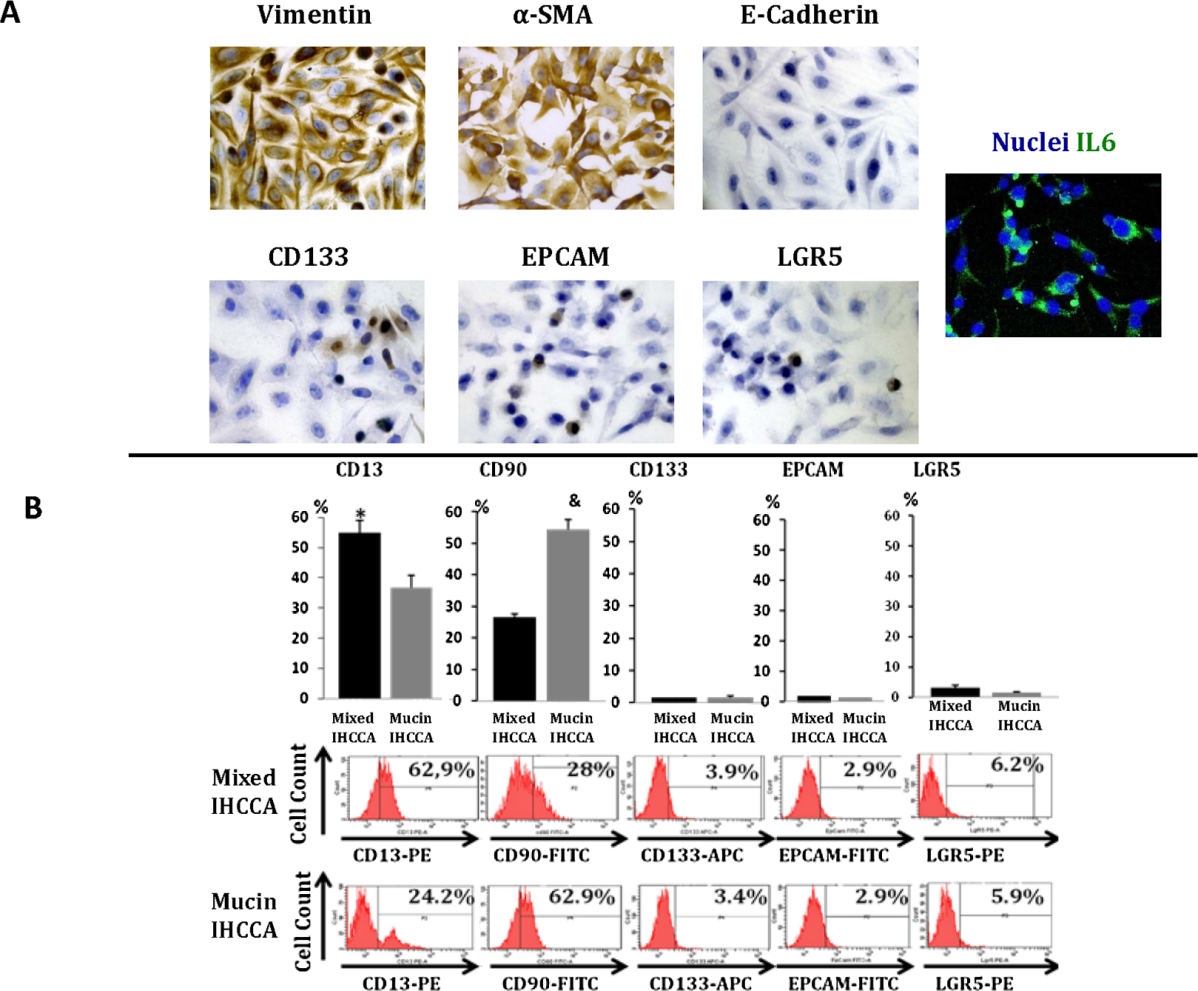
Characterization of mixed- and mucin-IHCCA primary cultures. (A) Immunohistochemical and immunofluorescence analyses of mixed- and mucin-IHCCA primary cultures for Vimentin, α-SMA, E-Cadherin and the “epithelial” cancer stem cell markers CD133+, EpCAM+, LGR5+ and for IL6. A diffuse positivity for mesenchymal markers (Vimentin, α-SMA) and IL6 was observed while E-Cadherin was virtually negative and less than 5% cells where positive for the “epithelial” cancer stem cell markers. Representative experiment of N = 12 independent staining performed in separate primary cultures. (B) Flow cytometry analyses of primary cultures of mixed- and mucin-IHCCA (20–30 passages), labeled with anti-CD13, anti-CD90, anti-EpCAM, anti-CD133, and anti-LGR5 antibodies. Bar graphs and representative plots. Cells positive for CD13 and CD90 largely predominated with respect to CD133, EpCAM and LGR5. CD13+ cells predominated in mixed-IHCCA with respect to mucin-IHCCA, while the opposite was found for CD90+ cells. Mean ± SD of N = 18 independent experiments. * = p< 0.05 vs mucin-IHCCA; & = p< 0.01 vs mixed-IHCCA.

Primary cultures prepared from human mixed- and mucin-IHCCA specimens were characterized for mesenchymal, epithelial and CSC surface markers by IH, FC and RT-PCR. As shown in Fig 1A, after 20–30 passages, most cells expressed mesenchymal markers (vimentin, αSMA) and IL6, were negative for E-Cadherin and CK-19 and rarely (< 5%) positive for “epithelial” CSC markers CD133, EpCAM, LGR5. In addition, most cells expressed the markers of EMT trait (Snail, TWIST, not shown). By FC (Fig 1B) cells positive for the mesenchymal CSC marker CD90 or for CD13, a marker of quiescent CSCs [2–4], largely predominated with respect to “epithelial” CSC markers, CD133, EpCAM and LGR5. When mixed- and mucin-IHCCCA primary cultures were compared, CD13+ cells predominated in mixed-IHCCA while the opposite was found for CD90+ cells (Fig 1B). These date were confirmed by RT-PCR (Table 3), where the expression of mRNA for vimentin and CD90 largely predominated with respect to CD133, EpCAM and LGR5, and mRNA for CD13 was more expressed in mixed-IHCCA while vimentin and the other CSC markers in mucin-IHCCA.

**Table 3:** RT-PCR analysis of Vimentin and cancer stem cell surface markers.

	VIM	CD90	CD13	CD133	EPCAM	LGR5
Mixed-IHCCA	4.3±0.36	0.49±0.002	0.05±0.0009	5.7*10^−3^±3.6*10^−5^	6.4*10^−5^±1.1*10^−5^	5.4 *10^−7^±3.8*10^−8^
Mucin-IHCCA	7.2±0.4*	0.95±0.0075*	0.024±0.0011*	4.3*10^−2^±2.6*10^−3^ *	7.5*10^−5^±1.2*10^−5^	5.8*10^−6^±7.1*10^−7^ *

mRNA relative expression of Vimentin (VIM), CD90, CD13, CD133, EpCAM and LGR5 in primary cultures from mixed-intrahepatic (Mixed-IHCCA) or mucin-intrahepatic (Mucin-IHCCA) human cholangiocarcinoma, normalized versus the reference gene GAPDH. mRNA for CD13 was more expressed in mixed-IHCCA while vimentin, CD90, CD133 and LGR5 predominated in mucin-IHCCA. Gene expression was measured in quadruplicate. Mean ± SD of N = 12 experiments.

* = p< 0.05.

### Effects of Chemotherapeutics on Cell Proliferation and Apoptosis

**Fig 2:**
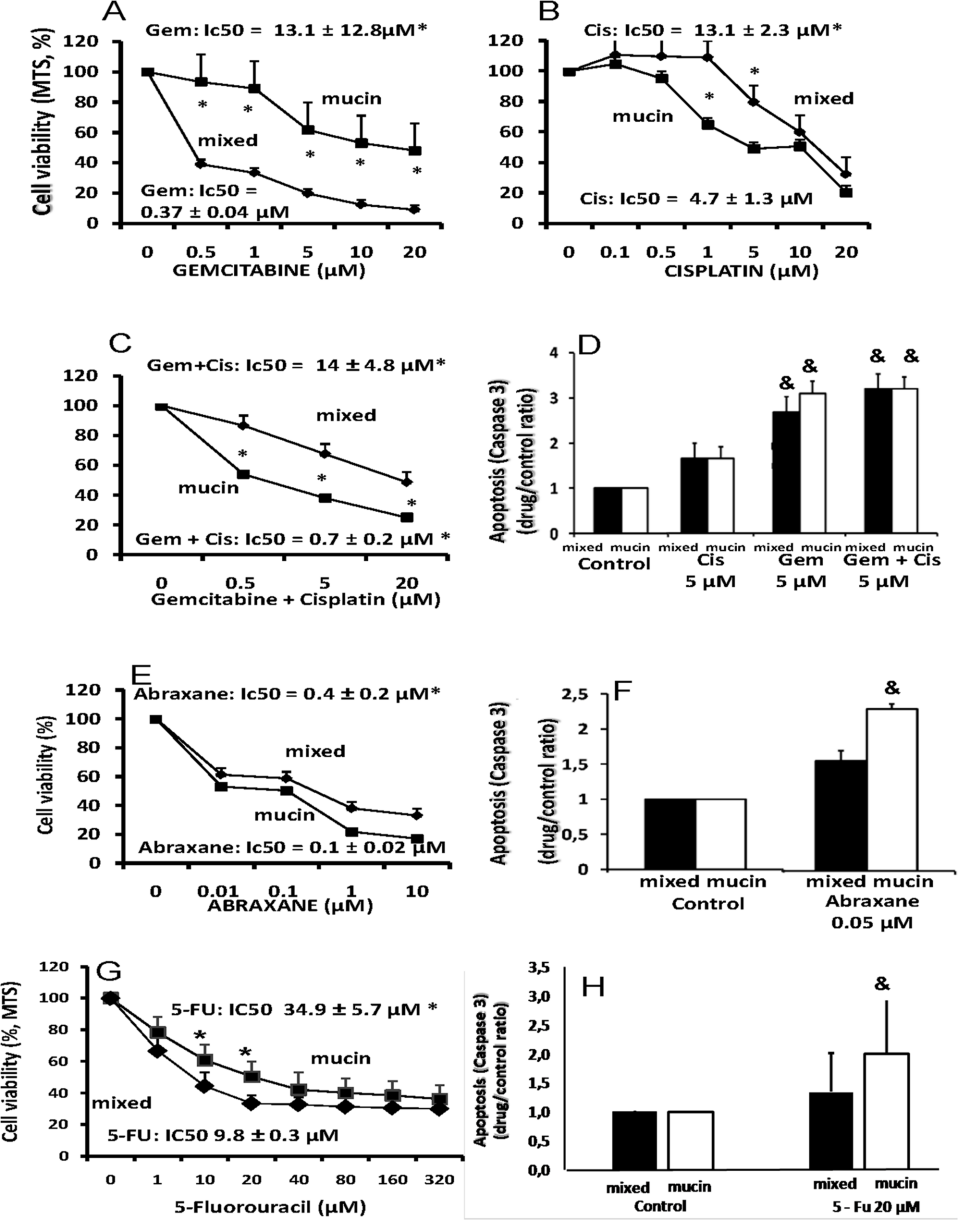
Effects of chemotherapeutics (Gemcitabine, Cisplatin, Abraxane) on proliferation and apoptosis of mucin- and mixed-CCA primary cultures. Cell proliferation was evaluated by MTS assay and results were expressed as percentage with respect to controls considered equal to 100. Apoptosis was evaluated by measuring caspase-3 activity and expressed as ratio between drug-treated and control cells. MTS and caspase-3 were measured 72 hours after incubation with the tested drug. Gemcitabine was much more active in inhibiting cell proliferation (A) (MTS assay) in mixed-IHCCA than mucin-IHCCA primary cultures. Cisplatin (B), in contrast, was more active against mucin-IHCCA. With respect to the two drugs alone, the Gemcitabile-Cisplatin combination induced a higher inhibition of cell proliferation (C) in mucin-IHCCA but not in mixed-IHCCA. Gemcitabine but not Cisplatin significantly enhanced Caspase-3 activity (D) without differences between mucin- and mixed-IHCCA and, the combination Gem+Cis does not further enhances the apoptotic effects of Gemcitabine alone. Abraxane showed a significant inhibitory effect on cell proliferation (E) in both mixed- and mucin-IHCCA primary cultures, although the effect on mucin-IHCCA was predominant (p< 0.05). Abraxane induced a significant increase of apoptosis only in mucin-IHCCA (F). 5-FU slightly inhibited cell proliferation (G) with a more significant effect on mixed- than mucin-IHCCA (p<0.05). 5-FU induced a significant increase of apoptosis only in mucin-IHCCA (H). *p< 0.05 mixed vs mucin. & = p< 0.05 vs controls. Mean ± SD of N = 5–7 independent experiments.

Gemcitabine and Cisplatin are the standard of care for CCA [1]. Fig 2A shows how Gemcitabine was much more active in inhibiting cell proliferation (MTS assay) in mixed-IHCCA (IC_50_ = 0.37 ± 0.04 μM) than mucin-IHCCA primary cultures (IC_50_ = 13.1 ± 12.8 μM, p< 0.01). Cisplatin, in contrast, was more active (Fig 2B) against mucin-IHCCA (IC_50_ = 4.7 ± 1.3 μM) than mixed-CCA (IC_50_ = 13.1 ± 2.3 μM, p< 0.01). With respect to the two drugs alone, the Gemcitabine-Cisplatin combination induced a higher inhibition of cell proliferation (Fig 2C) in mucin-IHCCA (IC_50_ = 0.7 ± 0.2 μM) but not in mixed-IHCCA (Gem+Cis; IC_50_ = 14 ± 4.8, Fig 1). As shown in Fig 2D, at 5 μM, Gemcitabine but not Cisplatin significantly enhanced apoptosis (Caspase 3, p< 0.01 vs controls) without differences between mucin- and mixed-IHCCA primary cultures while, the combination Gemcitabine+Cisplatin does not further enhances the apoptotic effects of Gemcitabine alone.

The microtubule inhibitor, Abraxane (paclitaxel protein-bound formulation) [8], showed (Fig 2E) a significant inhibitory effect on cell proliferation in both mixed- (IC_50_ = 0.4 ± 0.2 μM) and mucin-IHCCA (IC_50_ = 0.1 ± 0.02 μM) primary cultures, although the effect on mucin-IHCCA was predominant (p< 0.05). Abraxane (0.05 μM) induced a significant increase of apoptosis only in mucin- but not mixed-IHCCA (Fig 2F).

5-FU showed (Fig 2G) a slight inhibitory effect on cell proliferation in both mixed- (IC_50_ = 9.8 ± 0.3 μM) and mucin-IHCCA (IC_50_ = 34.9 ± 5.7 μM) primary cultures, although the effect on mixed-IHCCA was predominant (p< 0.05). 5-FU (20 μM) induced apoptosis but only in mucin-IHCCA (Fig 2H).

### Effects of Molecular Targeted Agents on Cell Proliferation and Apoptosis

#### -Sonic Hedgehog (Hg) pathway inhibitors

**Fig 3:**
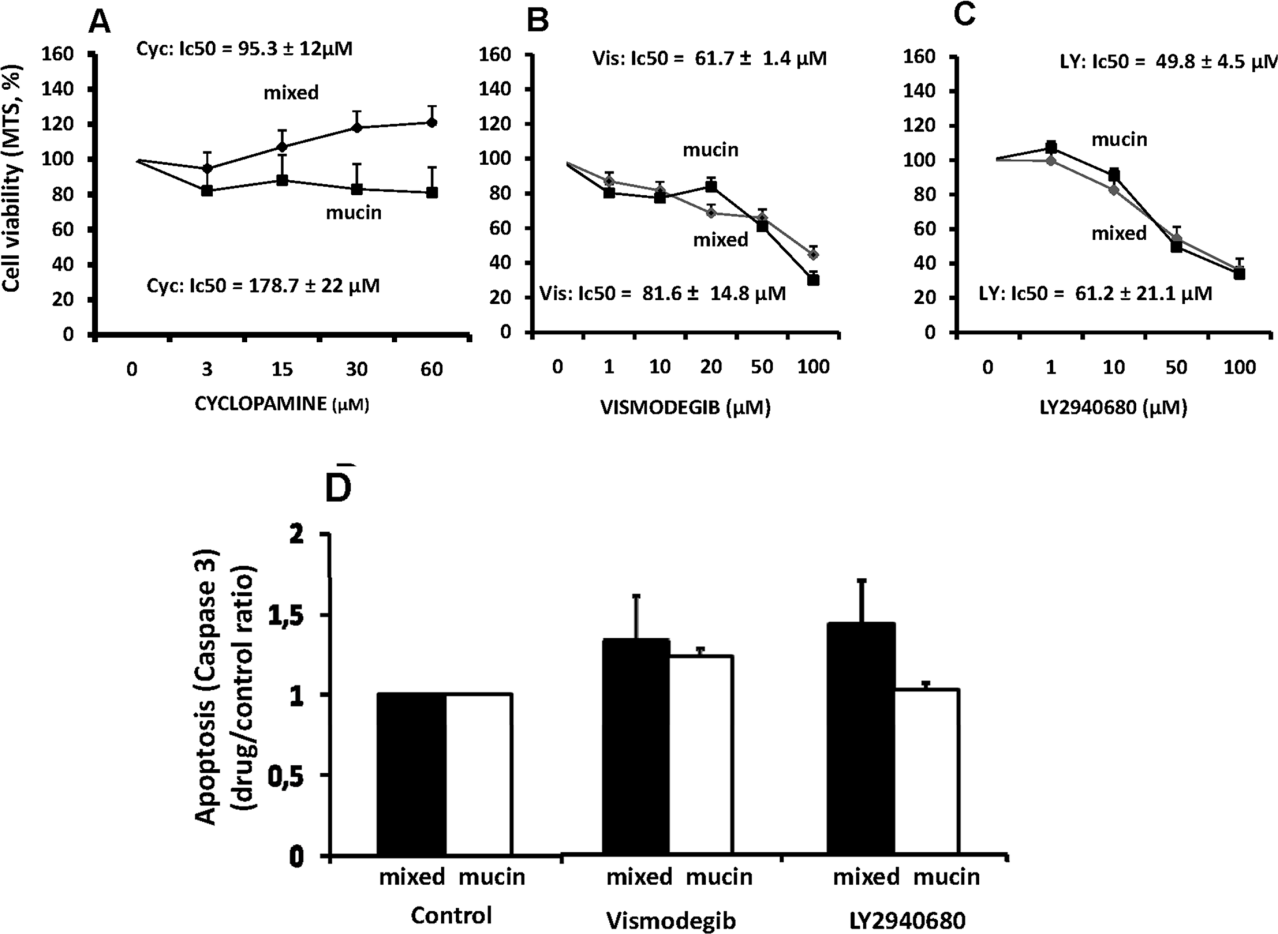
Effect of Sonic Hedgehog (Hg) pathway inhibitors on proliferation and apoptosis of mucin and mixed CCA primary cultures. Cyclopamine (A) displayed no effect on cell proliferation in primary cultures of both mucin- and mixed-IHCCAs. In contrast, Vismodegib (B) and LY2940680 (C), showed a slight inhibitory effect on cell proliferation in both CCAs subtypes. Apoptosis was unaffected by Vismodegib or LY2940680. N experiments = 5–7.

Cyclopamine, a natural occurring alkaloid [9], displayed no effect on cell proliferation in primary cultures of both mucin- and mixed-IHCCAs (Fig 3A). In contrast, Vismodegib, a small-molecule antagonist of Smoothened [9] and LY2940680, a small-molecule antagonist of the smoothened receptor [9], showed a slight inhibitory effect on cell proliferation (Fig 3B and 3C) without differences between mucin- (IC_50:_ Vismodecib = 61.7 ± 1.4 μM, LY2940680 = 49.8 ± 4.5 μM) and mixed-CCA (IC_50:_ Vismodecib = 81.6 ± 14.8 μM, LY2940680 = 61.2 ± 21.1 μM).

Apoptosis was unaffected by Vismodegib (50 μM) or LY2940680 (20 μM) in primary cultures of mucin- and mixed-IHCCA (Fig 3D)

#### -Tyrosine kinase inhibitors

**Fig 4:**
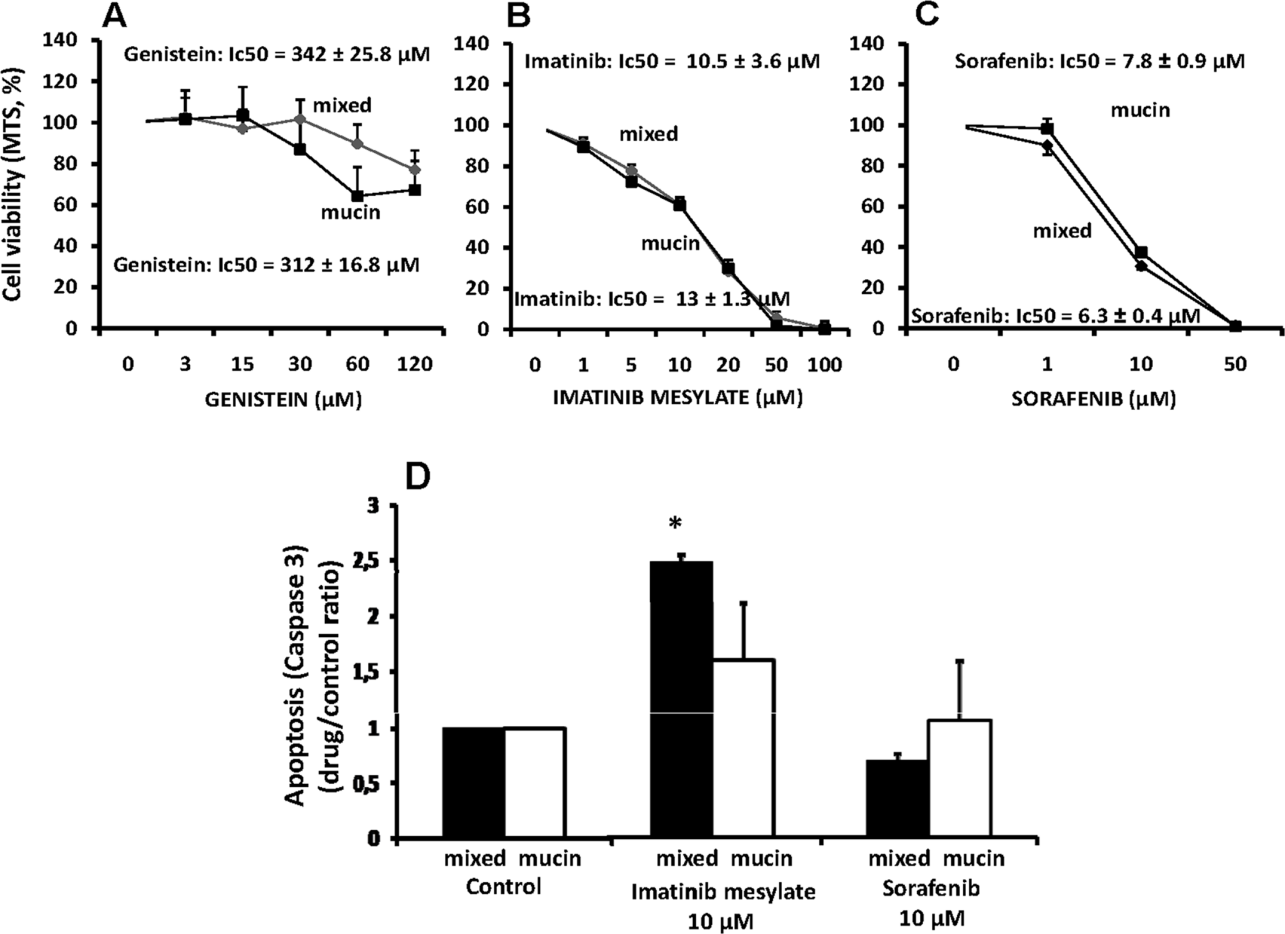
Effect of tyrosine kinase inhibitors on proliferation and apoptosis of mucin and mixed CCA primary cultures. Genistein showed no effect on cell proliferation (A). Imatinib mesylate (B) and Sorafenib (C) inhibited cell proliferation in mucin-IHCCA and mixed-IHCCA but only the former enhance apoptosis (D) and only in mixed-IHCCA. *p< 0.05 vs controls. N experiments = 5–7.

We tested the following tyrosine kinase inhibitors: Genistein that is a broad spectrum tyrosine kinase inhibitor [10]; Imatinib Mesylate that is a c-kit tyrosine kinase inhibitor also acting as aspecific anti-CD90+ cells [11], and; sorafenib that is a multi-targeted tyrosine kinase receptor inhibitor [12]. Primary cultures of mucin- and mixed-IHCCA were resistant to genistein (Fig 4A) but they were equally sensitive (inhibition of cell proliferation) to Imatinib Mesylate (IC_50:_ mucin-IHCCA = 10.5 ± 3.6 μM; mixed-IHCCA = 13.0 ± 1.3 μM, Fig 4B) and Sorafenib (IC_50:_ mucin-IHCCA = 7.8 ± 0.9 μM; mixed-IHCCA = 6.3 ± 0.4 μM; Fig 4C).

Apoptosis was significantly enhanced only by Imatinib Mesylate (10 μM) and only in mixed-IHCCA (Fig 4D).

#### -Epidermal growth factor receptor (EGFR) antagonists

**Fig 5:**
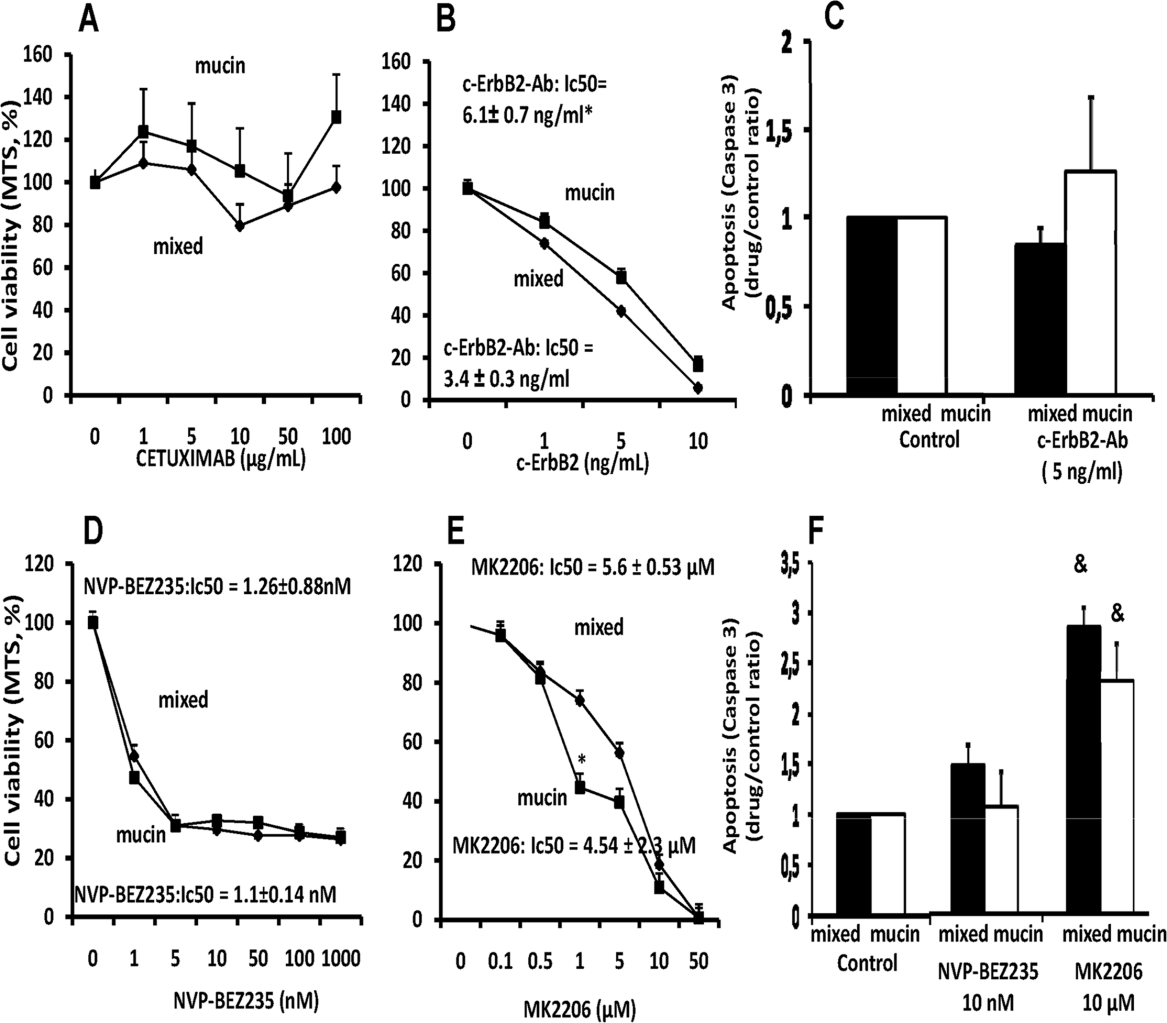
Effect of Epidermal growth factor receptor (EGFR) antagonists and PI3-kinase/AKT inhibitors on proliferation and apoptosis of mucin and mixed CCA primary cultures. The EGFR antagonist, cetuximab (A), had no effect on cell proliferation neither on mucin- nor in mixed-IHCCA. In contrast, the c-erbB2 blocking antibody (B) inhibited proliferation in both mucin- and mixed-IHCCA with a predominant effect on the latter. Cetuximab and the c-erbB2 blocking antibody showed no effect on cell apoptosis (C). *p< 0.05 mucin vs mixed, N experiments = 5–7. The PI3-kinase/AKT inhibitors, NVP-BEZ235 (D) and MK2206 (E) showed strong inhibitory effect on cell proliferation in both mucin- and mixed-IHCCA primary cultures, but only MK2206 enhanced apoptosis (F) without differences between mucin-IHCCA and mixed-IHCCA. *p< 0.05 mucin vs mixed, & = p< 0.05 vs controls. N experiments = 5–7.

Cetuximab, a chimerical monoclonal EGFR IgG1 antibody, that blocks the binding of EGF or other ligands to EGFR, thus, inhibiting ligand-induced receptor phosphorylation (11), had no effect neither on mucin- nor mixed-IHCCA (Fig 5A). In contrast, the c-erbB2 blocking antibody was active against both mucin-IHCCA (IC_50_ = 6.1 ± 0.7 ng/ml) and mixed-IHCCA (IC_50_ = 3.4 ± 0.3 ng/ml), but with a slight predominant effect on the latter (p< 0.05, Fig 5B). Cetuximab and the c-erbB2 blocking antibody (Fig 5C) showed no effect on cell apoptosis.

#### -PI3-kinase/AKT inhibitors

NVP-BEZ235, a dual PI3K and mTOR inhibitor [13], showed a very strong inhibitory effect, at nanomolar concentration, on cell proliferation in both mucin-IHCCA (IC_50_ = 1.1 ± 0.14 nM) and mixed-IHCCA (IC_50_ 1.26 ± 0.88 nM) primary cultures (Fig 5D). Also the allosteric AKT inhibitor, MK2206, inhibited cell proliferation (Fig 5E) in primary cultures of mucin-IHCCA (IC_50_ = 4.5 ± 2.3 μM) and mixed-CCA (IC_50_ 5.6 ± 0.53 μM) but at a significant lower extent in comparison with NVP-BEZ235.

As far as apoptosis is concerned, only the AKT inhibitor, MK2206, but not NVP-BEZ235, induces significant apoptotic effects, at 10 μM, without differences between mucin-IHCCA and mixed-IHCCA (Fig 5F).

#### -MEK 1/2 inhibitor

**Fig 6:**
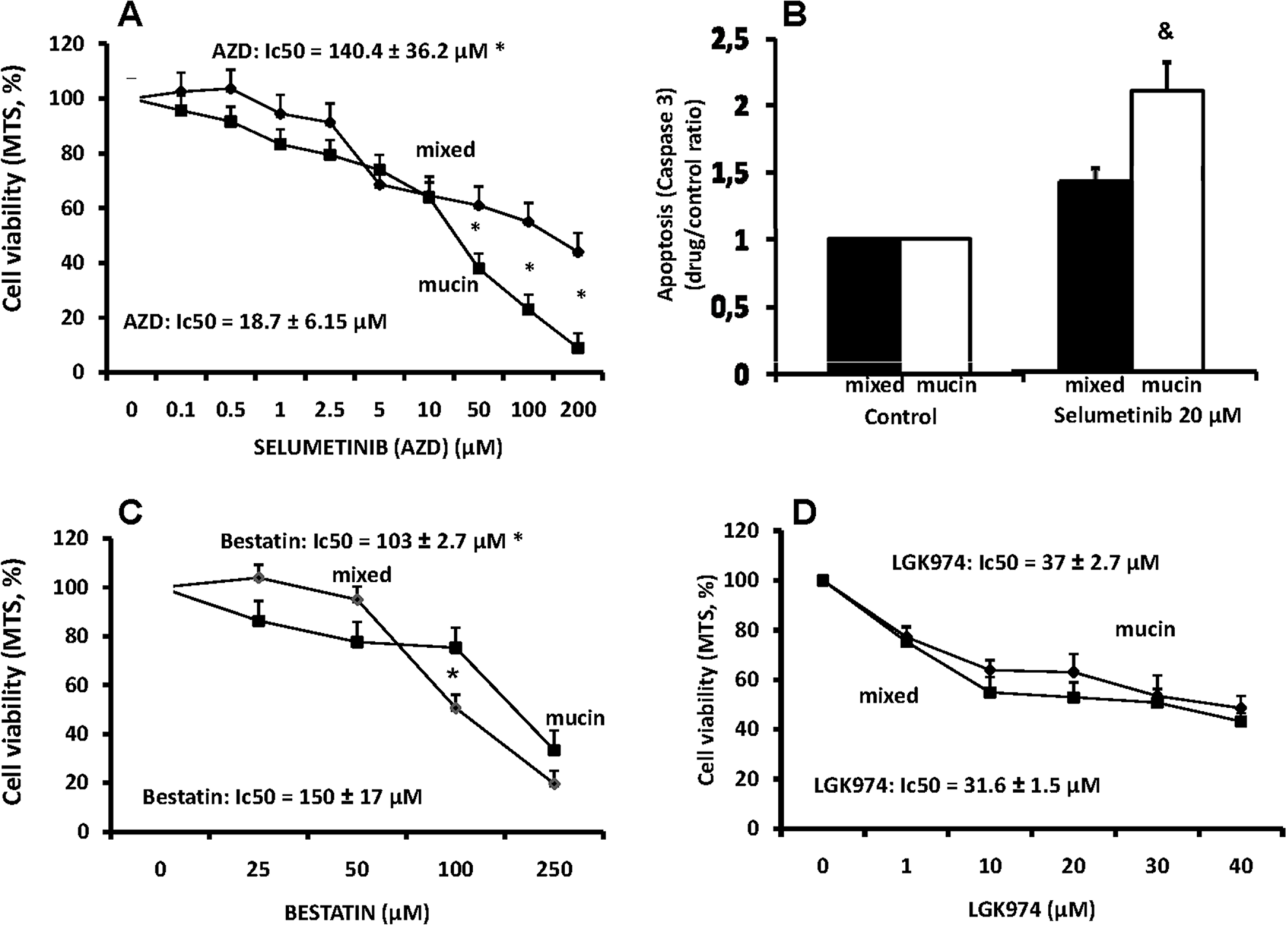
Effect of MEK 1/2 inhibitor, Aminopeptidase-N inhibitor and Wnt inhibitor on proliferation and apoptosis of mucin and mixed CCA primary cultures. The MEK 1/2 inhibitor, selumetinib (AZD6244) (A), inhibits cell proliferation only at high concentrations and with a predominant effect on mucin-IHCCA. Apoptosis (B) was enhanced only in mucin-IHCCA. *p< 0.05 mucin vs mixed, & = p< 0.05 vs controls. N experiments = 5–7. The aminopeptidase-N inhibitor, bestatin (C), showed an inhibitory effect on cell proliferation that predominated in mixed-IHCCA. *p< 0.05 mucin vs mixed, N experiments = 5–7. The Wnt inhibitor, LGK974, (D) starting from 10 μM concentration inhibited proliferation of both mucin- and mixed-IHCCA, N experiments = 3–5.

The selective, ATP uncompetitive, MEK 1/2 inhibitor, Selumetinib (AZD6244) [14] inhibits cell proliferation only at high concentrations and with a predominant effect on mucin-IHCCA (IC_50_ = 18.7 ± 6.1 μM) with respect to mixed-IHCCA (IC_50_ 140.4 ± 36.2 μM, p< 0.05; Fig 6A). Apoptosis was significantly enhanced by Selumetinib (20 μM) only in mucin-IHCCA (Fig 6B).

#### -Aminopeptidase-N inhibitor

The aminopeptidase-N inhibitor, bestatin, has been largely tested in a large spectrum of cancer cells, as CD13 inhibitor [15]. However, in both mucin- and mixed-IHCCA primary cultures, the inhibitory effect of bestatin on cell proliferation was limited with an IC_50_ of 103 ± 2.7 μM for mucin-IHCCA and of 150± 17 μM for mixed-IHCCA (p< 0.05, Fig 6C). No effect on apoptosis was seen at 100 μM (not shown).

#### - Wnt signaling Inhibitor

Recent evidence suggests that enhanced Wnt signaling sustains CCA progression and that targeting Wnt signaling could represent a potential therapeutic strategy [16]. We tested LGK974, a potent and specific small-molecule porcupine inhibitor. Porcupine is a membrane-bound O-acyltransferase that is required for palmitoylation of Wnt ligands, a necessary step in the processing of Wnt ligand secretion [17] Fig 6D shows how LGK794 induced, already at 10 μM concentration, an inhibitory effect on cell proliferation that was similar for mixed- (IC50 = 31.6 ± 1.5 μM) and mucin-IHCCA (37 ± 2.7 μM, p< 0.05). No significant effect on apoptosis was seen.

### *In Vivo* Sensitivity of Human Subcutaneous Xenografts to NVP-BEZ235 and Abraxane

**Fig 7:**
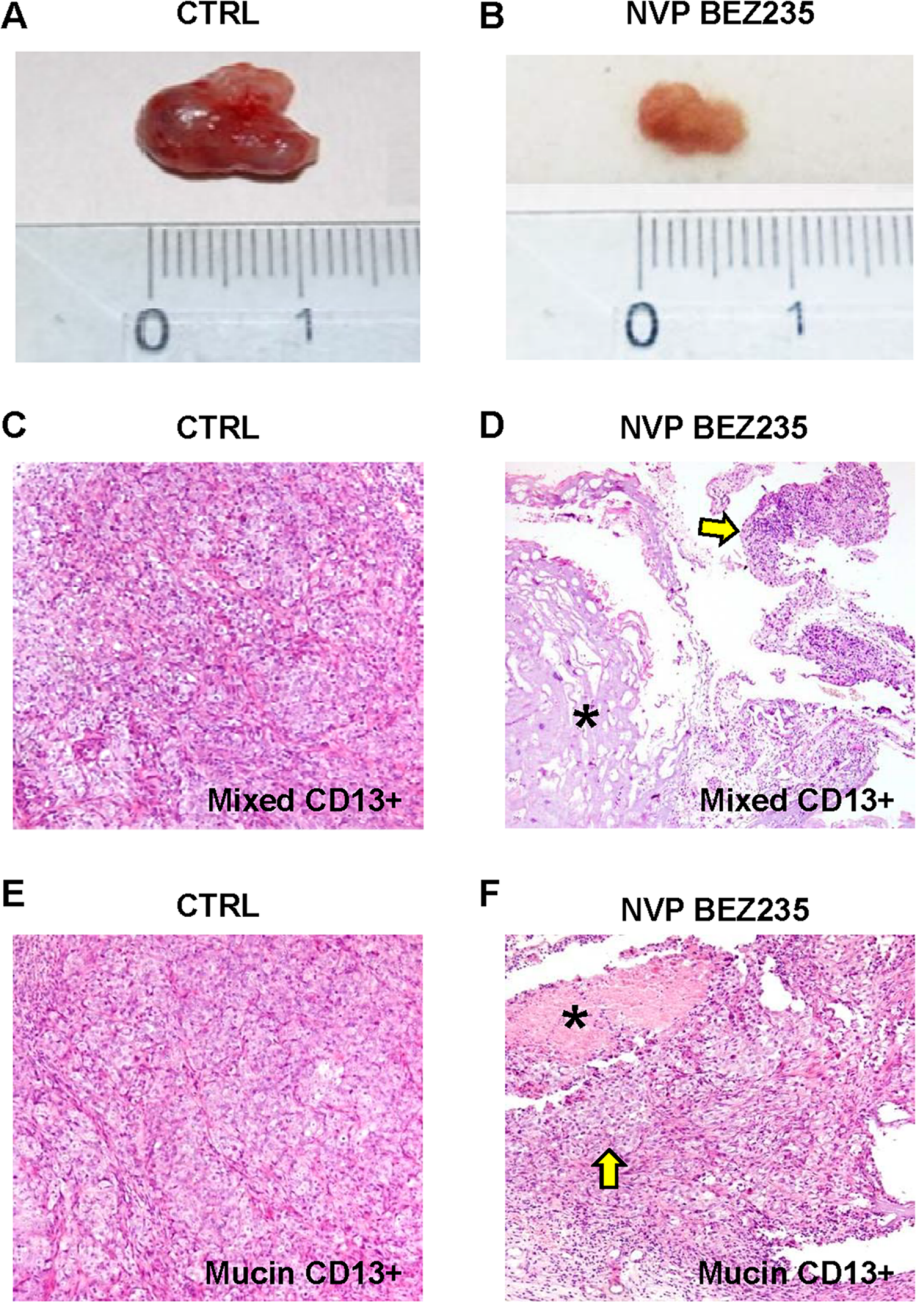
Effect of NVP-BEZ235 on subcutaneous human tumor xenografts. In control mice (CTRL), the tumor volume of subcutaneous xenografts increased during four weeks of observation (A) while in mice treated for 2 weeks with NVP-BEZ235 (B) it remains almost stable. At the histo-pathological evaluation (Hematoxylin-Eosin), in control mice (CTRL), subcutaneous masses arisen after the implantation of CD13+ spheroids from mixed- (C) or mucin-IHCCA (E) cultures, were composed of densely packed tumor cells. Original Magnification(OM): 10x. C, E). In mice treated with NVP-BEZ235, subcutaneous xenografts arisen from CD13+ spheroids, immunosorted from mixed-IHCCA cultures (D) were characterized by extensive necrosis (asterisk) and few tumoral cells (arrow); necrosis was less extended in masses arisen after the implantation of CD13+spheroids immunosorted from mucin-IHCCAs (F, asterisk = necrosis, arrow = tumoral cells). OM: 10x (D, F).

CD13+ or CD133+ spheroids prepared from primary cultures of human mucin- or mixed-IHCCA were subcutaneously injected in male NOD/SCID mice. After 2 weeks, when the tumors’ volume averaged 500 mm^3^, mice were treated by gavage with NVP-BEZ235 or Abraxane dissolved in PBS, while control mice received PBS only. At the fourth week, after 2 weeks of treatment, the volume of the masses were re-evaluated. In control mice, CD133+ and CD13+ tumor xenografts increased significantly, respectively from 550 ± 50 mm^3^ to 1400 ± 100 mm^3^ (p< 0.05), and from 590 ± 55 mm^3^ to 1450 ± 76 (p< 0.05, Fig 7A). No differences between mixed- and mucin-IHCCA were observed. In contrast, in mice treated with NVP-BEZ235 or Abraxane, the tumor volume for CD133+ spheroids remained almost stable (NVP-BEZ235: 790 ± 100 mm^3^ for mixed-IHCCA and 690 ± 100 mm^3^ for mucin-IHCCA; Abraxane: 730 ± 86 mm^3^ for mixed-IHCCA and 790 ± 96 mm^3^ for mucin-IHCCA, p< 0.05 vs controls). Similar findings were obtained when CD13+ spheroids were implanted, since the tumor volume remained almost stable following the treatment with NVP-BEZ235 (740 ± 100 mm^3^ for mixed-IHCCA and 650 ± 94 mm^3^ for mucin-IHCCA, Fig 7B, p< 0.05 vs controls) or Abraxane (670 ± 90 mm^3^ for mixed-IHCCA and 720 ± 85 mm^3^ for mucin-IHCCA, p< 0.05 vs controls). At the histo-pathological evaluation (H&E, Fig 7C–7F) in control mice, tumor xenographts were composed of densely packed tumor cells (Fig 7C mixed-IHCCA, Fig 7E mucin-IHCCA) while in mice treated with Abraxane or NVP-BEZ235 (Fig 7D mixed-IHCCA, Fig 7F mucin-IHCCA) necrotic areas were seen within the tumor mass.

## Discussion

The main findings of our study, performed in primary cultures of mucin- and mixed- IHCCA subtypes, indicate that: i) among chemotherapeutics, Gemcitabine and the Gemcitabine-Cisplatin combination were more active in inhibiting cell proliferation in mixed-CCA while Cisplatin or Abraxane were more effective against mucin-CCA, where abraxane was also able to induce apoptosis. 5-FU exerted a slight inhibitory effect on cell proliferation, predominating in mixed-IHCCA, and also induced apoptosis but only in mucin-IHCCA; ii) among Hg inhibitors, Ciclopamine was ineffective while LY2940680 and Vismodegib showed slight effects on proliferation but not on apoptosis; iii) the tyrosine kinase inhibitor, Genistein was completely ineffective while either Imatinib Mesylate or Sorafenib showed significant inhibitory effects on proliferation; iv) the MEK 1/2 inhibitor, Selumetinib, was active in inhibiting proliferation only against mucin-CCA while the aminopeptidase-N inhibitor, Bestatin was more active against mixed-CCA; v) the c-erbB2 blocking antibody was more active against mixed- than mucin-IHCCA while, Cetuximab failed to show significant effects; vi) either mucin- or mixed-IHCCA showed high sensitivity to PI3-kinase inhibitors and particularly to NVP-BEZ235 that markedly inhibited cell proliferation at nanomolar concentrations and, also *in vivo* blocked tumor progression. The main results of our study, based on experiments performed in primary cultures, indicate that mucin- and mixed-IHCCA display a different drug sensitivity. Specifically, Cisplatin, Abraxane and the MEK 1/2 inhibitor, Selumetinib are more active against mucin-IHCCA while, Gemcitabine, the Gemcitabine-Cisplatin combination, 5-FU, the c-erbB2 blocking antibody and the aminopeptidase-N inhibitor, bestatin, act better against the mixed-IHCCA subtype. Remarkably, both IHCCA subtypes showed a specific sensitivity to the PI3K/AKT inhibitors and in particular to the dual PI3-kinase/mTOR inhibitor, NVP-BEZ235, that both *in vitro* and *in vivo* exerts marked antiproliferative effect at very low concentrations.

The current standard of care for unresectable IHCCA is the Gemcitabine/Cisplatin regimen [18, 19] that has been recently considered to be also cost-effective with respect to Gemcitabine alone [20]. However, this treatment is absolutely unsatisfactory since ensure only a few month improved survival [18,19] and, therefore, alternative treatments are demanding. Unfortunately, CCA is scarcely responsive to chemotherapeutics for different reasons including its desmoplastic nature and the high expression of drug extruding pumps [1]. In addition, we have recently realized that CCA is highly enriched with CSCs suggesting this cancer as a disease of stem cells that are typically resistant to chemotherapeutics [5]. Very few phase I/II clinical trials evaluated the effects of molecular targeted agents, alone or in combination with gemcitabile or cisplatin [4]. In all these studies no discrimination between mucin- and mixed-IHCCA subtypes has been provided. Indeed, large morphologic, biologic and clinical differences exist between the mucin- and mixed-CCA subtypes [5,6]. From a clinical point of view, the mixed-subtypes is more frequently associated with liver cirrhosis, appears macroscopically as a mass-forming with a peripheral location and progress with less lymphatic and perineural invasion and with a better prognosis with respect to the mucin-CCA subtype [5]. In the previous manuscript [6], we demonstrated the large *in situ* differences in terms of CSC composition and EMT markers between the two CCA subtypes. Also primary cultures showed large differences since CD90+ cells predominated in mucin-CCA while CD13+ cells in the mixed–CCA subtype. In spite of these clinical and patho-biologic differences, no report investigated the drug-sensitivity of the two CCA subtypes and, clinically, the need of a different therapy has been so far completely neglected. We used primary cultures of mixed- and mucin-CCA to test drug sensitivity and to calculate IC50. These cultures are characterized by the predominance of cells expressing mesenchymal markers and EMT trait. We firstly demonstrated the different sensitivity of the two CCA subtypes to chemotherapeutics since Gemcitabine and the Gemcitabine-Cisplatin combination were more active against mixed-CCA while Cisplatin or Abraxane were more effective in inhibiting cell proliferation in mucin-CCA primary cultures. Abraxane is an albumin bound formulation of paclitaxel where albumin favors drug internalization and indeed, according to a recent report, this formulation showed promising results in advanced pancreatic cancer [21]. This is consistent with the present findings showing a preferential effects on mucin-IHCCA, since a number of biologic similarities exist between mucin-CCA and the adenocarcinoma of pancreatic ducts [22]. However, *in vivo*, in subcutaneous xenografts, Abraxane showed a similar inhibitory effect on the growth of both mucin- and mixed-IHCCA.

A critical point, in the era of targeted therapies, is the scarcity of basic or preclinical studies evaluating molecular targeted therapies in relation with biologic or genetic CCA signatures. Among the molecular target agents, we firstly tested the Hg signaling inhibitors since different studies suggested how the Hg signalling pathways are of relevance for CCA biology [23, 24]. The expression of Hg components, for example, was associated with the progression and metastasis in IHCCA [23, 24]. However, canonical Hg signaling requires cilia but CCA cells do not express cilia [25]. Nonetheless, CCA cells exhibit non-canonical Hedgehog signaling with may influence chemotaxis despite impaired cilia expression [25]. This non-canonical Hedgehog signaling pathway appears to contribute to CCA progression, thereby, supporting a role for Hedgehog pathway inhibition in human CCA [25]. We tested ciclopamine, the clinically approved small-molecule antagonist of Smoothened, Vismodegib, and of its receptor, LY2940680. In our experiments, CCA primary cultures were completely resistant to ciclopamine and slightly sensitive to LY2940680 and Vismodegib in terms of inhibited cell proliferation. Recently, by profiling transcriptomes in surgically resected samples, therapeutic targets for tyrosine kinase inhibitors have been identified [26]. Among the tyrosin kinase inhibitors, Genistein, a broad spectrum inhibitor [10], failed in our hands to influence cell proliferation of both CCA subtype primary cultures. In contrast, Sorafenib, a multiple kinase inhibitor [10] played antiproliferative effects without influencing apoptosis. It is noteworthy that the concentrations demonstrated to be effective in our study correspond to the steady-state sorafenib plasma levels of 15–20 μM measured in patients administered with approved dosages of this drug [27]. In clinical trials testing Sorafenib, Dealis et al. [28] showed a controlled disease in one third of patients with advanced CCA while, Bengala et al. [29] showed an improved progression free survival only in patients with better conditions. We finally tested Imatinib Mesylate that acts not only as c-kit-R inhibitor [9] but also, as recently demonstrated [6], as aspecific blocker of CD90+ CSCs [11]. Since CD90+ cells represent a predominant CSC subpopulation in mucin-IHCCA, our result should merit investigation in clinical trials. To this regard, in a phase II study [30], the disease control rate of 26 CCA patients was 58% and this looks promising.

EGFR and/or ErbB2 are overexpressed in human and rodent CCA cells and EGFR has been implicated with CCA pathogenesis [31, 32]. EGFR overexpression was found as an independent prognostic factor for survival and a risk factor for tumor recurrence after resection in intrahepatic CCA and, associated with tumor progression and invasion in extrahepatic CCA [31]. According to this literature, EGFR has been considered an attractive target for CCA therapy [31]. In 2010, by investigating the efficacy and safety of cetuximab in combination with gemcitabine and oxaliplatin for first-line treatment of biliary tract cancer, Gruenberger et al. [33] reported objective response in 63% of patients. Unfortunately, these promising findings have not been confirmed. In fact, in a non-comparative, open-label, randomised phase-2 trial, the addition of cetuximab to chemotherapy (cisplatin or oxaliplatin plus gemcitabine) in advanced biliary cancers, although well tolerated, failed to enhanced the activity of chemotherapeutics [34], In our experiments, cetuximab failed to influence cell proliferation or apoptosis. In contrast, the c-erbB2 blocking antibody was active against both CCA subtypes in inhibiting cell proliferation but without effects on apoptosis. To this regard, a dramatic response to Trastuzumab, the antibody targeting HER2 receptor molecule was described in a patient with EGFR2–positive metastatic CCA [35].

Signaling mediated by PI3K and mTOR play a critical role in CCA cell proliferation, as well as in modulating authophagy and drug resistance [36–38]. Increased activation of PI3K/AKT signaling was reproducibly observed in CCA tissues and the expression of mTOR was significantly correlated with metastasis [36–38]. Interestingly, PTEN suppression by loss of expression or inactivation by phosphorylation was observed in the majority of patients [36]. Therefore, PI3K/mTOR inhibition is currently considered a potential effective therapeutic strategy for CCA [36–38]. To this regard, NVP-BEZ235 a novel and potent imidazo[4,5-c] quinolone derivative, that dually inhibits both PI3K and mTOR kinases, effectively inhibited CCA cell growth and migration and significantly induced G1 arrest without apoptosis induction, although increased autophagy was observed [36]. The same conclusion was raised by Ewald F. et al. [38] showing, in preclinical studies, how combined targeting of mTOR and AKT using RAD001 and MK-2206 inhibited the growth of CCA cell lines. In our experiments, NVP-BEZ235, that is now entering phase I/II clinical trials, played at nanomolar concentrations marked antiproliferative effects against both mucin- and mixed-CCA without enhancing apoptosis. Notably, also *in vivo*, in subcutaneous xenografts, NVP-BEZ235, blocked the progression of mucin- and mixed-IHCCA. Also the pan-AKT inhibitor, MK2206, exerted antiproliferative affects and, this compound also enhanced apoptosis. Thus our findings support future clinical trials testing PI3k/mTOR inhibitors.

Selumetinib is an allosteric inhibitor of MEK1 and MEK2 phosphorylation of ERK and of interleukin-6 secretion, the latter playng a major role in modulating CCA cell proliferation [1]. In a phase-II study, in patients with metastatic biliary tract cancer [14], 12% of patients had a confirmed objective response and, 68% of these patients experienced stable disease. In our study, Selumetinib showed inhibitory effects on proliferation and activation of apoptosis; these effects being predominant in mucin-IHCC subtype.

We also tested Bestatin, a CD13+ CSC antagonist, that showed antitumoral activity in different cancers [15]. We found a preferential effect of Bestatin on mixed-IHCCA primary cultures and this is consistent with the predominance of CD13+ CSCs in mixed CCA subtypes.

Finally, we tested a Wnt signalling inhibitor, since recent evidence highlighting the role of Wnt signaling in sustaining CCA growth and progression [16]. Starting form 10 μM, LGK974, inhibited by approx. 40–50% cell proliferation without differences between Mucin- and mixed-IHCCA.

In conclusion, our findings indicate that other than biologically different, mucin and mixed-CCA subtypes are also different as far as the sensitivity to chemotherapeutics and targeted-agents is concerned. Future therapeutic strategies triggering IHCCA should take in consideration these differences and, therefore, discrimination between the two subtypes is fundamental also for therapeutic decisions.
